# Slingshot-1L, a cofilin phosphatase, induces primary breast cancer metastasis

**DOI:** 10.18632/oncotarget.19855

**Published:** 2017-08-03

**Authors:** Chen Chen, Yusufu Maimaiti, Shen Zhijun, Liu Zeming, Guo Yawen, Yu Pan, Huang Tao

**Affiliations:** ^1^ Department of Breast and Thyroid Surgery, Union Hospital, Tongji Medical College, Huazhong University of Science and Technology, Wuhan 430022, P.R. China; ^2^ Department of General Surgery (Research Institute of Minimally Invasive), People’s Hospital of Xinjiang Uygur Autonomous Region, Urumqi 830000, P.R. China; ^3^ Clinical Laboratory, The Third People’s Hospital of Hubei Province, Wuhan 430000, P.R. China

**Keywords:** breast cancer, cofilin, slingshot, membrane protrusionm, actin dynamics

## Abstract

Slingshot (SSH) is a member of the conserved family of cofilin phosphatases that plays a critical role in cell membrane protrusion and migration by transforming inactive phosphorylated cofilin to an active form. SSH-like protein 1 (SSH-1L) expression is detected in various types of tumors; insulin induces the phosphatases activity of SSH-1L in a phosphoinositide 3-kinase-dependent manner. However, little is known about the expression and role of SSH-1L in breast cancer. Here, we analyzed 295 human breast cancer tissue specimens for SSH-1L expression by immunohistochemistry. The correlation between SSH-1L level and patients’ clinical characteristics was analyzed with Pearson’s χ^2^ test. The function of SSH-1L was evaluated by gene knockdown and quantitative real-time polymerase chain reaction detection of cofilin expression in MDA-MB-231, MCF-7, and SK-BR-3 human breast cancer cell lines. SSH-1L expression was detected in 88.1% of tissue specimens by immunohistochemistry and was strongly associated with increased metastasis and mortality. Loss of SSH-1L expression decreased the nonphosphorylated, active form of cofilin in SK-BR-3 and MDA-MB-231 cell lines, which was associated with reduced cell motility. Accordingly, SSH-1L/cofilin signaling played a critical role in primary breast cancer metastasis and was a potential therapeutic target for breast cancer treatment.

## INTRODUCTION

Cell migration plays a major role in tumor invasion and metastasis; it is initiated by membrane protrusion, which is mediated by the dynamics of actin filaments [1–2] that assemble as a network at the leading edge of migrating cells [3]. Cofilin stimulates actin depolymerization and severance of actin filaments at the protruding ends, thereby promoting their rapid turnover and determining the direction of cell movement [4–5]. Cofilin activity is regulated by its phosphorylation status, and is negatively regulated by serine/threonine kinases, LIM kinases, and testis-specific protein kinase via phosphorylation at Ser-3 [6–8]. Phospho-cofilin is dephosphorylated and reactivated by Slingshot (SSH), a member of the conserved family of protein phosphatases [9–10].

In mammalian cells, SSH phosphatases are encoded by three genes (*SSH-1*, *-2*, and *-3*). SSH-like proteins 1, 2, and 3 (SSH-1L, SSH-2L, and SSH-3L, respectively) exhibit distinct tissue expression patterns, subcellular distribution, and activities [9, 11]. SSH-1L expression fluctuates over the course of the cell cycle *in vitro* [12]. Insulin-stimulated MCF-7 cells exhibit increased SSH-1L activity and cofilin dephosphorylation, which is abrogated by phosphoinositide 3-kinase (PI3K) inhibition [13–15]; moreover, in these cells SSH-1L accumulates in protrusions where active cofilin is concentrated and directly binds insulin receptor substrate-4 [16]. The interaction of SSH-1L with F-actin determines its activation and is required for the chemotactic response of cells [6, 8]. The mitotic kinase Aurora (Aur)-A, which induces mammary cell migration, induces SSH-1L expression in breast cancer [17], implying that the regulation of cell migration by Aurora-A may be achieved by modulation of SSH-1L expression.

Breast cancer is one of the most common malignancies worldwide with poor prognosis [18–19]. Breast cancer metastasis has been extensively [20–21] and accumulating evidence implicates cofilin signaling as a major determinant of this process [22–23]. However, there is still relatively little information on the role of the cofilin regulatory factor SSH-1L in breast cancer.

We addressed this in the present study by investigating SSH-1L expression in human breast cancer tissue and its correlation with clinical features such as metastasis and mortality. We also examined the effect of SSH-1L knockdown on cofilin phosphorylation and breast cancer cell motility and the underlying mechanisms. The results indicate that SSH-1L stimulates breast cancer cell migration via dephosphorylation of cofilin, thereby promoting metastasis. Thus, targeting SSH-1L is a potential therapeutic strategy for preventing breast cancer progression.

## RESULTS

### SSH-1L expression in human breast cancer tissue is correlated with lymph node metastasis and poor prognosis

**Figure 1:**
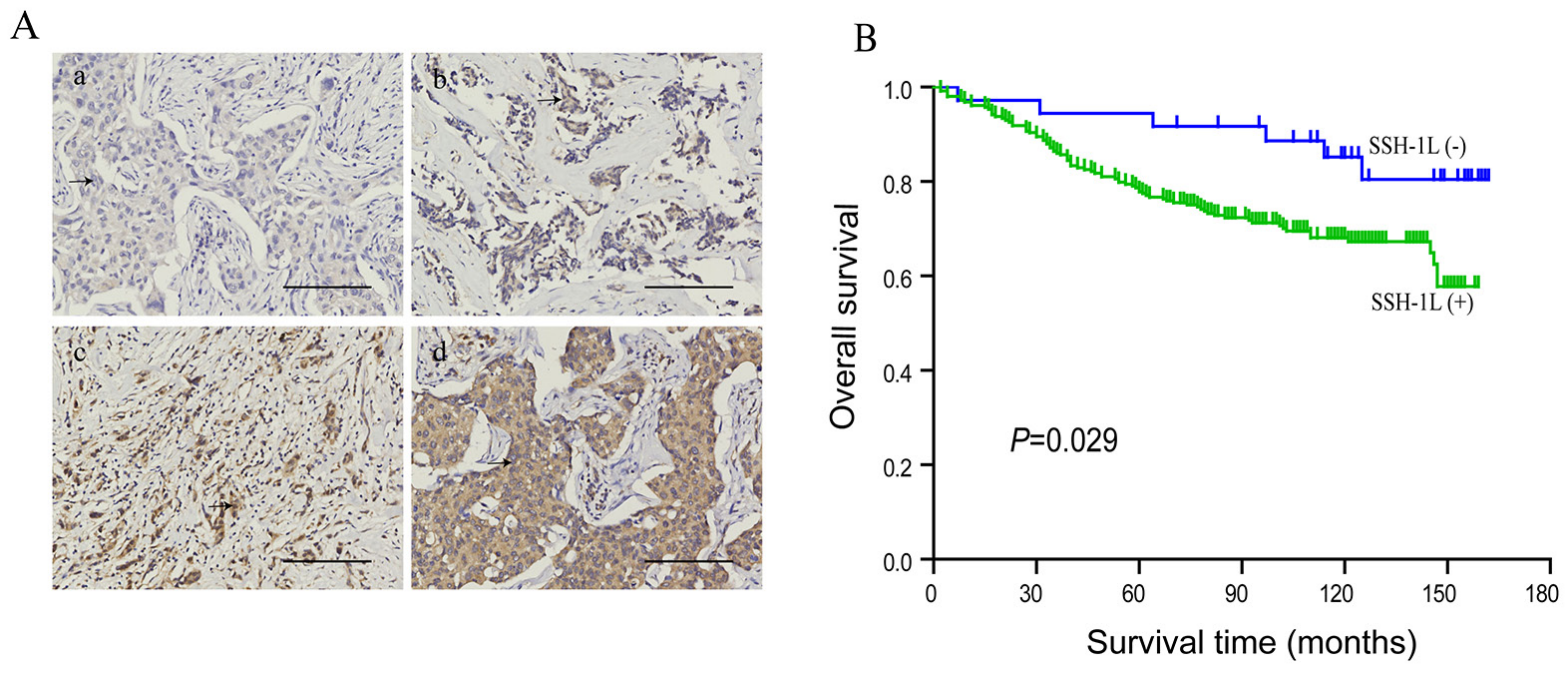
**(A)** SSH-1L expression in human breast cancer, illustrating representative intensity scores as 0(a); 1(b); 2(c); 3(d). All images were captured at the same magnification, arrow depict tumor cells, bar, 100μm. **(B)** Correlation of SSH-1L expression and survival in all patients, P<0.05 is considered significant.

A tissue microarray containing 295 human breast cancer patient specimens was used for immunohistochemical analysis. SSH-1L was detected in 260/295 samples. We established a standard protocol to define the intensity of cytoplasmic labeling, with each sample was graded on a scale of 0–3 (Figure 1A). We also investigated the correlation between SSH-1L expression and overall survival of patients. We found that patients who were negative for SSH-1L expression had higher survival rates than those who were positive (Figure 1B). In addition, SSH-1L expression, tumor stage, node stage, and TNM stage influenced the overall survival according to the log-rank test (Table 1). In the multivariate Cox regression analysis, SSH-1L expression was significantly associated with patient prognosis (P=0.038, 95% confidence interval: 1.052–5.633) (Table 2). In addition, lymph node metastasis rate was higher in patients who were positive for SSH-1L expression than in those who were negative (P=0.017; Table 3 ).

**Table 1:** Kaplan-Meier survival analysis of SSH-1L expression and other clinical pathologic parameters

Variable	Censored			P
	Total N	Survival	Ratio	
Age 1				0.778
<35	10	7	70.00%	
≥35	285	200	70.18%	
Age 2				0.936
<50	104	73	70.19%	
≥50	191	134	70.16%	
Pathology grade				0.812
1	41	27	65.85%	
2	228	162	71.05%	
3	26	18	69.23%	
T stage				0.004
1	74	58	78.38%	
2	188	132	70.21%	
3	33	17	51.52%	
N stage				0.004
0	134	103	76.87%	
1	82	60	73.17%	
2	58	33	56.90%	
3	21	11	52.38%	
TNM stage				0.001
0	1	1	100.00%	
1	38	31	81.58%	
2	168	127	75.60%	
3	88	48	54.55%	
SSH-1L				0.029
–	35	29	82.86%	
+	259	177	68.34%	
Her-2				0.588
–	226	159	70.35%	
+	69	48	69.57%	

**Table 2:** Multivariate Cox regression analysis of prognostic factors

	B	Exp(B)	SE	P	95%CI	
					Lower	Upper
Age	0.184	1.202	0.23	0.422	0.766	1.866
T stage	0.405	1.499	0.226	0.073	0.963	2.332
N stage	0.144	1.155	0.196	0.464	0.786	1.696
TNM stage	0.306	1.358	0.357	0.391	0.675	2.732
SSH-1L	0.889	2.434	0.428	0.038	1.052	5.633

**Table 3:** Correlation of SSH-1L expression and lymph node metastasis of breast cancer

Variable	Number of patients			P
	Total	Metastasis	Ratio	
SSHIL				0.017
−	36	14	38.89%	
+	259	146	56.37%	

### SSH-1L expression is associated with cofilin activity in breast cancer cells

**Figure 2:**
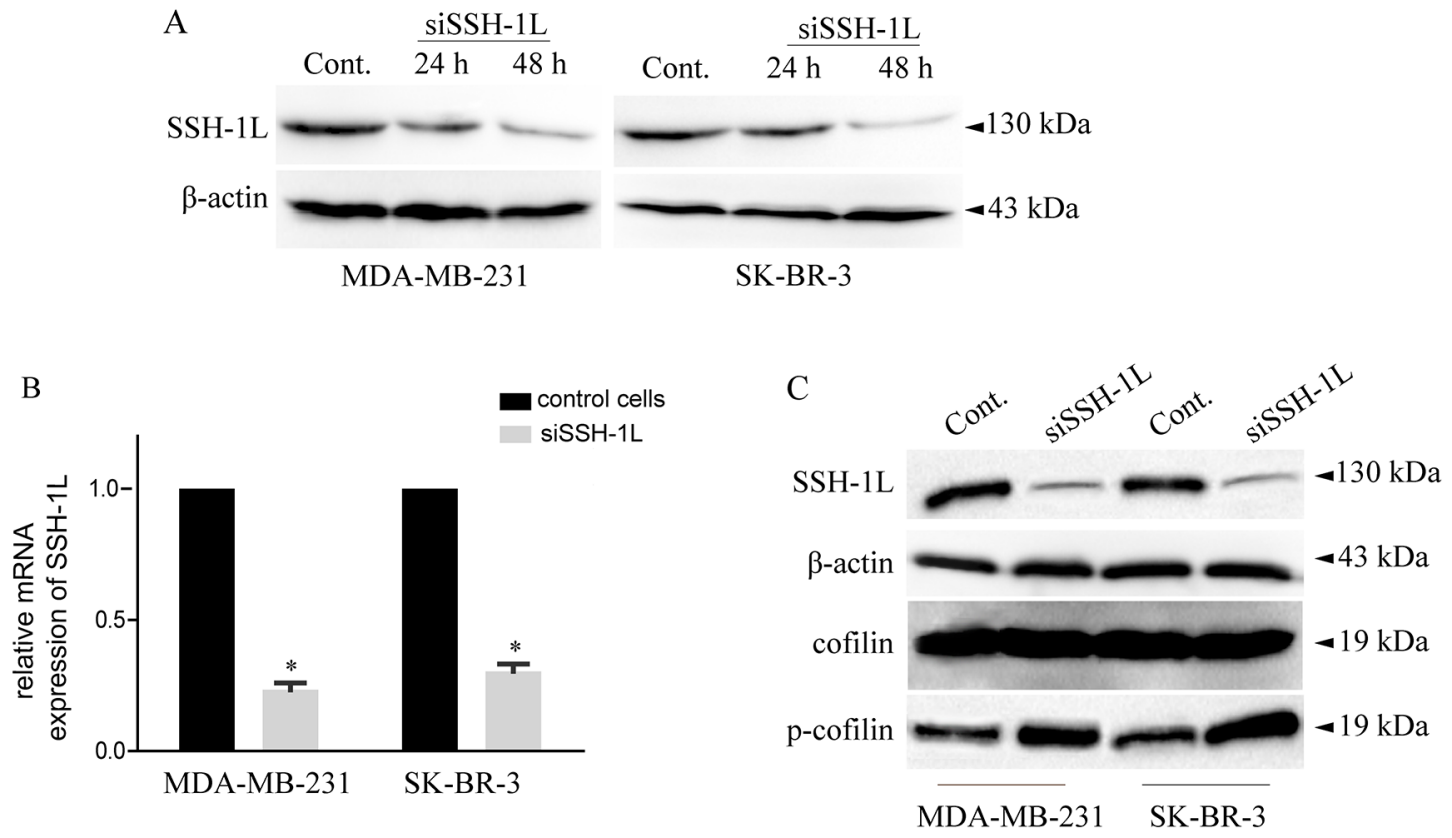
SSH-1L expression was detected in human breast cancer cell lines and loss of SSH-1L decrease the expression of cofilin and increase expression of p-cofilin **(A)** SSH-1L expression in different cell lines detected by western-blot, each cell line grouped by control, transfected with siSSH-1L after 24 h and 48 h. **(B)** Relative mRNA expression after knockdown of SSH-1L for 48 h in MDA-MB-231 and SK-BR-3 cells analyzed by RT-PCR. * P< 0.05. **(C)** The expression of SSH-1L, cofilin, p-cofilin, were detected in MDA-MB-231 and SK-BR-3 cells after knockdown of SSH-1L for 48h. The expression of actin was used to normalize the loading volume.

Cofilin is inactivated by phosphorylation at Ser-3 by LIM kinase and reactivated by dephosphorylation by SSH-1L [13]. This determines the invasive and metastatic phenotype of tumor cells [4, 22]; moreover, our previous work demonstrated that cofilin overexpression is associated with thyroid cancer cell migration [24]. In this study, we examined the role of SSH-1L on breast cancer cell migration and found that SSH-1L was up-regulated in breast cancer cell lines with high metastatic potential. In addition, siRNA-mediated knockdown of *SSH-1L* in SK-BR-3 and MDA-MB-231 cells increased cofilin phosphorylation in both cell lines (Figure 2B, 2C). These results suggest that SSH-1L expression is associated with cofilin activity in breast cancer.

### Loss of SSH-1L expression decreases breast cancer cell migration

**Figure 3:**
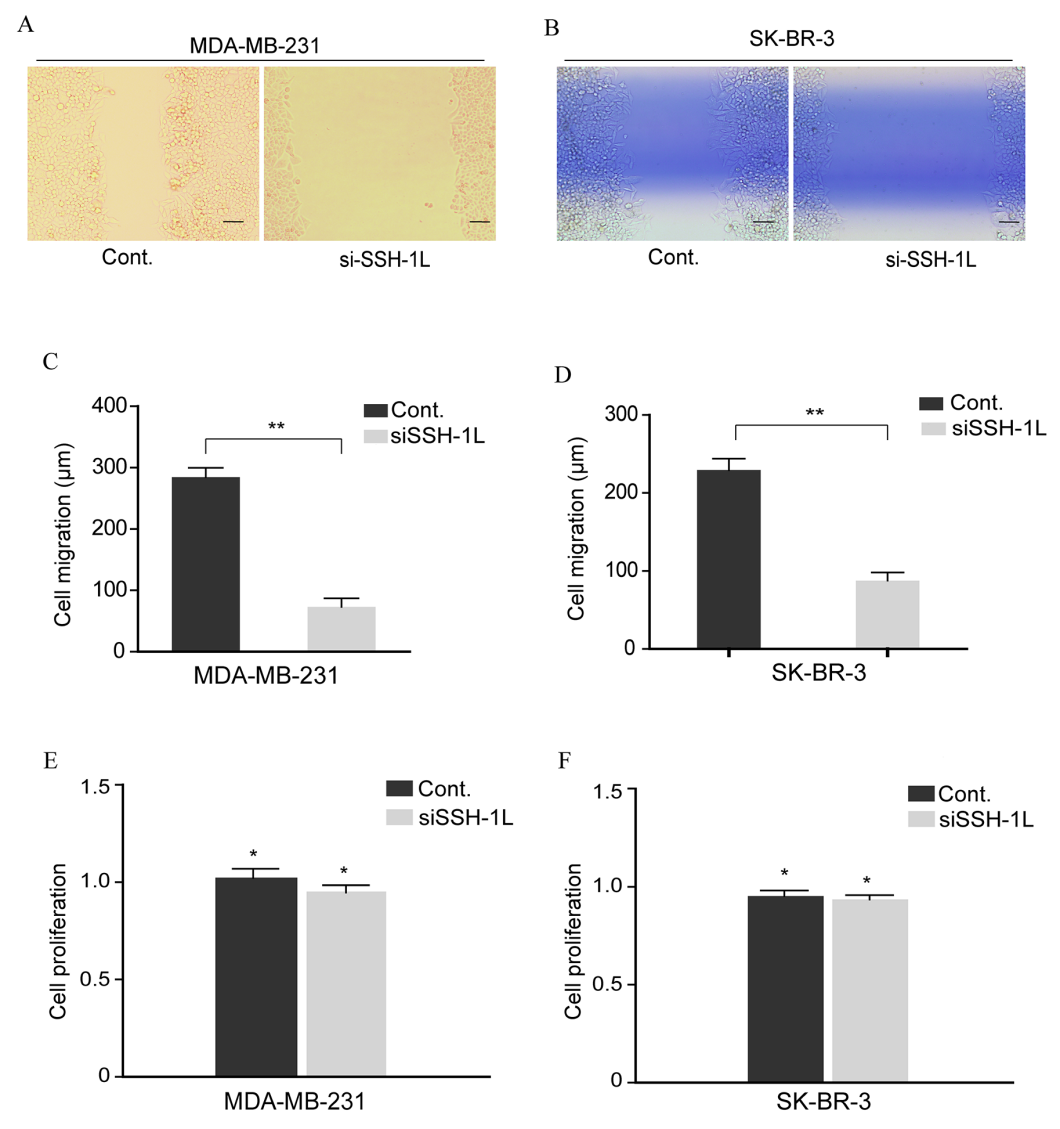
Inhibition of SSH-1L expression reduces breast cancer cell migration **(A)** and **(B)** Cell migration of MDA-MB-231 and SK-BR-3 were assessed by cell scratching assay. **(C)** and **(D)** Qualification of cell migration was analyzed by image J and data was performed by t-test, **, P< 0.01. **(E)** and **(F)** Relative cell proliferation was estimated by MTT after SSH-1L was knocked down for 72h, *, P<0.5.

The effect of SSH-1L knockdown on breast cancer cell migration was evaluated with the wound healing assay. After 24 h, loss of SSH-1L reduced the migration of SK-BR-3 and MDA-MB-231 cells relative to control cells (Figure 3A–3D). However, cell proliferation was unaltered by SSH-1L deficiency, indicating that the observed effect was not due to growth inhibition (Figure 3E, 3F). Thus, SSH-1L is specifically involved in breast cancer cell migration and may thereby regulate cell invasion and metastasis.

### Insulin activates SSH-1L/cofilin signaling in a PI3K-dependent manner

**Figure 4:**
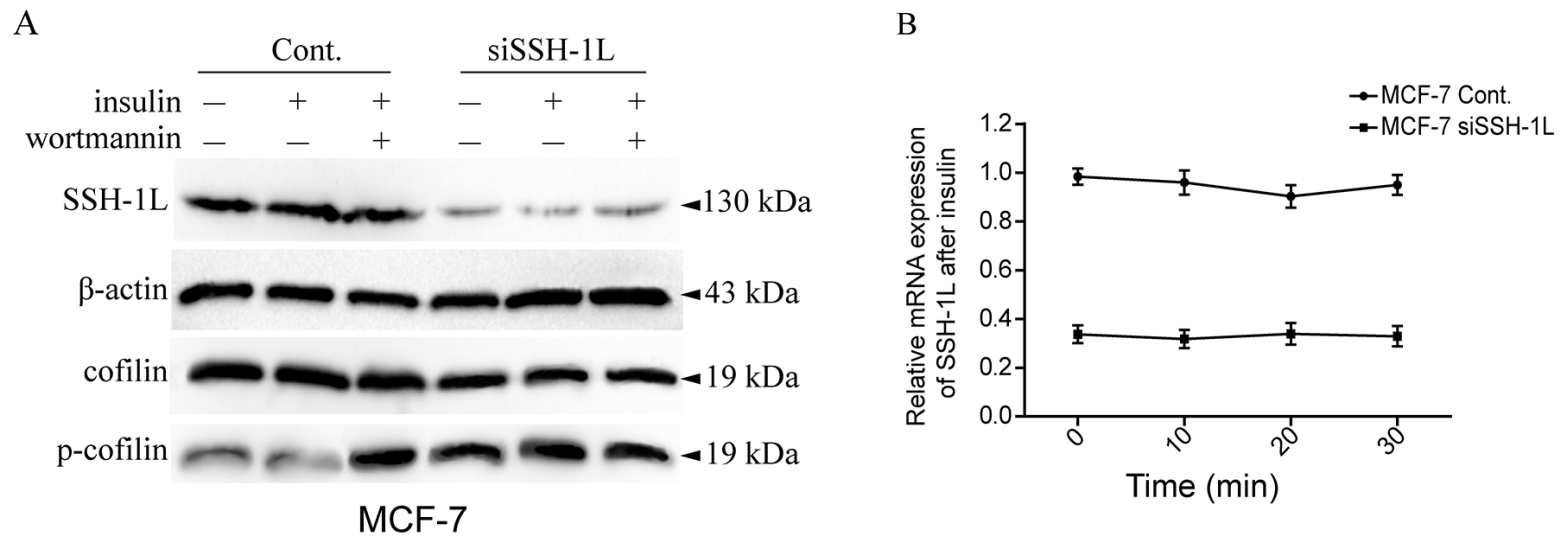
Insulin induce SSH-1L activation and cofilin dephosphorylation without altering the expression of SSH-1L in a PI3K-dependent manner **(A)** The expression and siSSH-1L groups were incubated with or without 10μg/ml insulin for 30 minutes, then the insulin (+) cells of each group were incubated with or without wortmannin, a PI3K inhibitor. **(B)** The relative mRNA expression of SSH-1L was detected by RT-PCR respectively at 0, 10, 20,30 minutes after incubation of MCF-7 cell with insulin.

Insulin induces the activation of LIMK1 and consequently of cofilin to regulate cell membrane protrusion. PI3K plays a critical role in cofilin dephosphorylation via SSH-1L activation [13]. We therefore investigated the role of insulin and the PI3K-dependence of SSH-1L/cofilin signaling in breast cancer cells and found that insulin treatment induced cofilin dephosphorylation without altering SSH-1L expression (Figure 4A, 4B). Cofilin phosphorylation was increased upon treatment with wortmannin or SSH-1L knockdown in the presence or absence of insulin (Figure 4A). These results demonstrate that insulin induces cofilin dephosphorylation by enhancing SSH-1L activity in a PI3K-dependent manner.

## DISCUSSION

In this study, we showed that SSH-1L was overexpressed in 88.1% of clinical breast cancer specimens, and that this predicted breast cancer lymph node metastasis and poor survival in patients. Loss of SSH-1L increased cofilin phosphorylation and inhibited cell migration in SK-BR-3 and MDA-MB-231 human breast cancer cell lines, indicating that SSH-1L regulates cell motility via cofilin dephosphorylation. In insulin-stimulated MCF-7 cells, increasedSSH-1L activation reduced cofilin phosphorylation, while SSH-1L knockdown abolished insulin-induced cofilin dephosphorylation. SSH-1L is a phosphatase that specifically dephosphorylates cofilin at Ser-3 [11] and thus plays a critical role in actin dynamics, which in turn modulate tumor metastasis and invasion [25]. The results presented here demonstrate for the first time that SSH-1L modulates breast cancer metastasis via regulation of cofilin signaling.

Cofilin is a member of the actin-depolymerizing factor/cofilin family of proteins [6, 26] that regulate the formation of the actin cytoskeleton [22]. Tumor invasion and metastasis—which is the main cause of cancer-related death—is directly associated with cofilin activity [23, 27]. Our previous work also showed that the phosphorylation status of cofilin is associated with survival in breast cancer patients [28]. Another study reported that the overall activation of the cofilin pathway determines the invasive and metastatic phenotype of tumor cells [29].

As a regulator of cofilin, SSH-1L affects tumor migration by altering the activity of cofilin/F-actin. The cofilin-phosphatase activity of SSH-1L is increased by binding to F-actin [30–32] via Trp-458 at the C-terminus of the phosphatase domain and the N-terminal LHK and C-terminal LKR motifs. A pleckstrin homology-like domain at the N-terminus of SSH-1L was also shown to be involved in F-actin binding and cofilin phosphatase activity [33].

In conclusion, our results reveal an important role for SSH-1L/cofilin signaling in breast cancer. These findings provide a basis for the development of drugs that target SSH-1L to prevent breast cancer progression and improve patient outcome.

## MATERIALS AND METHODS

### Tissue specimens and patients’ clinical information

The study population included 295 invasive breast cancer patients who underwent surgery as initial treatment between 2001 and 2008 at hospitals in Shanghai, Jiangsu, and Zhejiang. Patients provided written, informed consent for participation in the study at the National Engineering Center for Biochip at Shanghai. A diagnosis of breast cancer was confirmed based on postoperative pathological findings and followed standard guidelines among all these hospitals during the period. The following clinical characteristics were recorded for each patient: age; gender; tumor size; lymph node invasion; tumor-node-metastasis (TNM) stage; and estrogen and progesterone receptor and human epidermal growth factor receptor (HER)-2 status. Patients with breast cancer were followed from the date of surgery to the date of death or last follow-up.

### Immunohistochemistry

SSH-1L, cofilin, and Aur-A expression in clinical specimens was detected by immunohistochemistry using anti-SSH-1L (ab76943, 1:100) and -cofilin-1 (ab42824, 1:1500) antibodies (both from Abcam, Cambridge, MA, USA) and anti-Aur-A antibody (#14475, 1:50; Cell Signaling Technology, Danvers, MA, USA). The degree of immunoreactivity in the cytoplasm was evaluated by two experienced pathologists blinded to patients’ clinical information. Expression was graded as positive (high staining intensity, >5%) or negative (low staining intensity, 0–5%) [24]. The slides were scanned using a ScanScope scanner (Aperio, Vista, CA, USA), and images of representative areas were acquired using Image Scope software (Leica, Wetzlar, Germany).

### Cell culture

MDA-MB-231, MCF-7,and SK-BR-3 human breast cancer cell lines were purchased from the American Type Culture Collection (Manassas, VA, USA). MDA-MB-231 cells were maintained in L-15 medium (Gibco, Grand Island, NY, USA) supplemented with 10% fetal bovine serum (FBS; Gibco) at 37°C in a humidified incubator. MCF-7 cells were maintained in Dulbecco’s Modified Eagle’s medium (Gibco) supplemented with 10% FBS and 10mM estrogen (E2758; Sigma-Aldrich, St. Louis, MO, USA). SK-BR-3 cells were cultured in MyCoy’5a medium (Gibco) supplemented with 10% FBS at 37°C in a humidified atmosphere of 5% CO_2_- 5% air. Insulin was purchased from Sigma-Aldrich and wortmannin was obtained from Selleck Chemicals (S2578; Houston, TX, USA).

### Antibodies and quantitative real-time (qRT-)PCR primers

Antibodies specific for SSH-1L and cofilin-1 were purchased from Abcam (ab76943 and ab42824, respectively). Antibodies against Aur-A and phospho-cofilin-1 were from Cell Signaling Technology (#14475 and #3313, respectively; Danvers, MA, USA). Antibody against β-actin was purchased from Santa Cruz Biotechnology (sc-81178; Santa Cruz, CA, USA). Horseradish peroxidase(HRP)-conjugated anti-mouse and -rabbit antibodies were from Cell Signaling Technology (#7076 and#7078, respectively). The following sense and anti-sense primers were used for qRT-PCR: SSH-1L, 5’-GGAAGAATCGTCACCCAA-3’ and 5’-CAGGCGGTAGAAGAAAGG-3’; and β-actin, 5’-GTGGACATCCGCAAAGAC-3’ and 5’-TGGGTGCCAGGGCAGTGATC-3’.

### Short interfering (si)RNA-mediated knockdown of SSH-1L gene expression

An siRNA oligonucleotide (5′-ucgucacccaagaaagauatt-3′) was used to knock down *SSH-1L* expression, with *lacZ*-specific siRNA used as a negative control. Breast cancer cells were seeded in a 6-well flat-bottom plate (2×10^5^ per well). At 70% confluence, the culture medium was replaced and 30 min later, cells were transfected with SSH-1L or control siRNA using Lipofectamine 2000 (Invitrogen, Carlsbad, CA, USA) according to the manufacturer’s protocol.

### Western blotting

Cells were seeded in a 6-well plate until the number reached 1×10^6^ per well. The supernatant was discarded and cells were washed with phosphate-buffered saline before homogenization on ice for 30 min in radio immuno-precipitation assay buffer containing 1%phenylmethylsulfonyl fluoride. Cells were collected by scraping into sterile tube and centrifuged at 12,000 × *g* for 15 min at 4°C. The supernatant was collected and protein concentration was determined with the bicinchoninic acid assay (Thermo Fisher Scientific, Waltham, MA, USA). Equal amounts of protein were resolved by sodium dodecyl sulfatepolyacrylamide gel electrophoresis on a Mini-PROTEAN Tetra Cell (Bio-Rad, Hercules, CA, USA) and transferred to a 0.45-μm polyvinylidene difluoride membrane (Millipore, Billerica, MA, USA) that was blocked overnight with 5% skimmed milk diluted in Tris-buffed saline with0.1% Tween-20 (TBST) for 1 h followed by overnight incubation at 4°C with the primary antibodies. The membrane was washed three times with TBST and then incubated with HRP-conjugated secondary antibody for 1 h at room temperature. Protein bands were visualized using a chemiluminescence reagent (Goodbio Biotechnology, Wuhan, China). The blots were imaged with a Molecular Imager ChemiDoc XRSt (Bio-Rad) and ImageLab v.4.1 software (Bio-Rad) was used to determine the background-subtracted density of the bands.

### Cell scratching assay

Cells were seeded in a 6-well flat-bottom plate at 2×10^5^. Twenty-four hours later, cells were transfected with SSH-1L or control siRNA. Forty-eight hours later, cells were serum starved with 0.1 % FBS in EBM medium for 24 h. A scratch was made to the bottom of the well through the cells with a sterile 200 μl pipette tip. After washes to remove those that were non-adherent, the cells were cultured in EBM medium containing 0.1% FBS and examined at 0, 16 and 24h with a BZ-9000 epifluorescence microscope (Keyence, Itasca, IL, USA). Quantification was performed using ImageJ software (National Institutes of Health, Bethesda, MD, USA).

### Proliferation assay

Cells were seeded in 96-well plates at 2×10^3^ cells/well. Twenty-four hours later, cells were transfected with SSH-1L or control siRNA using Lipofectamine 2000 (Invitrogen, Carlsbad, CA, USA) according to the manufacturer’s protocol. Forty-eight hours later, cells were serum starved with 0.1 % FBS in EBM medium for 24 h; A proliferation assay was carried out with cell counting kit-8 (Sigma-Aldrich, St. Louis, MO, USA)following the protocol provided by the producer. Data are expressed as the mean±SD of quadruplicate values.

### Statistical analysis

Group means were compared with the Student’s t test and Pearson’s χ^2^ test was used to assess correlations between variables. The Cox regression proportional hazards model was used to estimate hazard ratios for lymph node metastasis based on Aur-A and CFL-1 expression. All statistical tests were two-sided and a P value <0.05 was considered statistically significant. Analyses were performed using SPSS v.19 software (SPSS, Chicago, IL. USA).
